# Barriers, Facilitators, and Training Preferences for HIV PrEP Among Physicians in the State of Louisiana, USA

**DOI:** 10.1007/s10900-026-01627-1

**Published:** 2026-08-20

**Authors:** Deborah Gurgel Smith, Patricia Pichilingue-Reto, Catherine G. Dean, Corey D. Smith, Luis Enrique Espinoza

**Affiliations:** 1 School of Health Professions and Sciences, Louisiana State University Health Sciences Center-Shreveport, 1501 Kings Highway, Shreveport, LA 71103 USA; 2 Department of Medicine, Louisiana State University Health Sciences Center-Shreveport, Shreveport, LA USA; 3 School of Medicine, Louisiana State University Health Sciences Center-Shreveport, Shreveport, LA USA; 4 Department of Internal Medicine, Louisiana State University Health Sciences Center-Shreveport, Shreveport, LA USA; 5 College of Nursing and Health Sciences, Texas A&M University - Corpus Christi, Corpus Christi, TX USA

**Keywords:** PrEP, HIV prevention, Physicians, Structural barriers, Prescribing practices

## Abstract

HIV pre-exposure prophylaxis (PrEP) is highly effective in preventing HIV infection, yet its implementation remains suboptimal in the Southern United States. This study examined barriers, facilitators, and training preferences related to PrEP implementation among physicians at an academic medical center in North Louisiana. A cross-sectional electronic survey was conducted between January 2024 and April 2025 among physician faculty, residents, and fellows. Participants reported perceived barriers and facilitators to PrEP implementation, preferred training topics and modalities, and demographic characteristics. Descriptive statistics summarized responses, and linear regression identified factors associated with physicians’ PrEP implementation training scores. The most commonly reported barriers were lack of provider training, insurance and cost-related concerns, lack of clinical protocols, staffing and time constraints, and limited knowledge of laboratory monitoring. Frequently identified facilitators included access to clinical protocols, peer support, pharmacist support, and electronic medical record (EMR) order sets. Physicians most frequently requested training on PrEP eligibility, side effects and toxicity, and contraindications, with in-person training as the preferred format. Physicians identified substantial educational and structural barriers to PrEP implementation despite strong support for institutional strategies that facilitate prescribing. Expanding physician education while integrating standardized protocols, pharmacist support, and EMR-based decision tools may strengthen PrEP implementation in high HIV-burden regions of the Southern United States.

**Supplementary Information:**

The online version contains supplementary material available at https://doi.org/10.1007/s10900-026-01627-1.

## Introduction

Despite advances in HIV prevention and treatment, the HIV/AIDS epidemic remains a significant concern for public health in the United States. According to the Centers for Disease Control and Prevention (CDC), an estimated 1.2 million individuals were living with HIV in the United States in 2022, and approximately 31,800 new HIV diagnoses were reported that year [1]. Although HIV affects all regions of the country, the burden is disproportionately concentrated in the Southern United States. In 2022, the South accounted for approximately 52% of new HIV diagnoses and nearly half of all individuals living with HIV nationwide [1]. Louisiana still has one of the highest HIV burdens in the country, ranking fourth among U.S. states for new HIV diagnoses. According to state surveillance data, nearly 24,000 people were living with diagnosed HIV as of June 2025 [2].

Pre-exposure prophylaxis (PrEP) has been shown to be a safe and effective medication for reducing HIV transmission, reducing the risk of sexual transmission of HIV by 99%, and transmission of HIV by injection drug use by 74% [3]. Despite its demonstrated effectiveness, PrEP uptake remains lower than needed in many high-burden communities. This gap is particularly evident in the Southern United States, where HIV incidence remains high, but PrEP utilization lags behind other regions of the country. In 2023, the South accounted for more than half of new HIV diagnoses nationwide but represented only 38% of PrEP users [4].

Multiple factors contribute to the underutilization of PrEP. At the patient level, barriers include limited awareness, HIV-related stigma, concerns about medication costs, and challenges accessing healthcare services [3]. At the physician and health-system levels, barriers include insufficient knowledge regarding PrEP prescribing, lack of confidence discussing sexual health and HIV prevention, uncertainty regarding clinical eligibility and monitoring requirements, and limited institutional infrastructure to support implementation [5]. Previous research has also indicated that physicians have expressed concerns about issues such as insurance coverage, medication costs, monitoring adherence, and the extra time needed for counseling and follow-up care [6].

Physicians are essential in increasing PrEP availability and enhancing the PrEP-to-need ratio in communities with high HIV burden. Research has shown that provider recommendation is among the most influential factors in increasing patient awareness, initiation, and sustained use of PrEP [3]. However, physicians frequently report inadequate training and limited institutional support for PrEP implementation, particularly in primary care settings and in regions with fewer HIV specialty resources [7, 8]. Identifying barriers, facilitators, and workforce training needs is therefore essential for developing effective implementation strategies and strengthening HIV prevention efforts in the Southern United States. Therefore, this study aimed to identify barriers and facilitators influencing PrEP implementation among physicians at an academic medical center in North Louisiana and to assess provider preferences regarding PrEP-related training. Understanding these factors may help guide future educational interventions, inform institutional implementation strategies, and support efforts to expand access to PrEP in high HIV-burden regions.

## Methods

### Study Design and Setting

This cross-sectional survey study was conducted between January 2024 and April 2025 among physicians affiliated with a public academic medical center and its teaching hospital in North Louisiana. Louisiana consistently ranks among the U.S. states with the highest HIV diagnosis rates, particularly within the Southern region, highlighting the need to strengthen institutional capacity for HIV prevention strategies, including pre-exposure prophylaxis (PrEP). The present analysis focused specifically on physician-reported barriers, facilitators, and training preferences related to PrEP implementation. The study was designed to evaluate implementation readiness and inform workforce development and training strategies within an academic healthcare setting.

### Participants and Recruitment

Eligible participants included physician faculty members, residents, and fellows actively engaged in direct patient care across clinical specialties. Participants were recruited using convenience sampling through institutional listserv email invitations, printed flyers posted in clinical and academic areas, and brief presentations during departmental grand rounds and morning reports. Recruitment materials included a Quick Response (QR) code and a direct hyperlink to the electronic survey, allowing physicians to complete the questionnaire using a computer, tablet, or mobile device. Upon accessing the survey, participants were presented with an informational letter describing the study purpose, the voluntary nature of participation, and the anonymous handling of responses. Participants provided electronic consent before proceeding.

Because no prior estimates were available regarding the prevalence of barriers and training needs among physicians in this setting, sample size calculations were based on an assumed proportion of 50%, which yields the most conservative estimate for cross-sectional studies [9]. Assuming a 95% confidence level and a margin of error of ± 10% points, the estimated minimum sample size was 96 participants. A total of 104 physicians completed the survey. After excluding incomplete responses via listwise deletion, the final analytic sample comprised 100 physicians.

## Measures

### Barriers to PrEP Implementation

Physicians were asked whether they had encountered specific barriers to prescribing PrEP. Five barrier domains were assessed: lack of provider training or education, lack of clinical protocols or guidelines, lack of knowledge of laboratory monitoring, staffing or time constraints, and insurance- or cost-related concerns. Responses were coded as binary variables (yes/no).

### Facilitators of PrEP Implementation

Physicians were asked to identify institutional supports that would facilitate PrEP implementation. Facilitator domains included access to PrEP protocols and guidelines, peer support from knowledgeable colleagues, pharmacist support for medication counseling and adherence monitoring, and electronic medical record (EMR) order sets with embedded PrEP guidance. Responses were coded as binary variables (yes/no).

### Training Preferences

Physicians identified PrEP-related topics for which they desired additional training. Six content areas were assessed: PrEP eligibility criteria, side effects and toxicity, contraindications, adherence counseling, behavioral risk assessment and sexual history taking, and sexual behavior counseling. Multiple responses were permitted. To quantify overall training demand, a PrEP implementation training score was calculated by summing the number of selected training topics (range: 0–6). Higher scores reflected greater perceived training needs and served as the primary outcome variable in regression analyses. Physicians also indicated preferred training delivery modalities, including in-person workshops, self-directed online courses, online learning resources, clinical consultation with HIV experts, webinars, and tele-mentoring. Each modality was coded as a binary variable.

### Physician Characteristics

Demographic and professional characteristics included age (continuous), gender, race/ethnicity, professional role (faculty, resident, or fellow), clinical specialty, and years in practice. Years in practice were categorized as < 5 years, 5–10 years, 11–20 years, and > 20 years.

### Data Management and Statistical Analysis

Survey data were collected electronically using REDCap (Research Electronic Data Capture), a secure web-based data management platform, and analyzed using IBM SPSS Statistics version 26.0. Descriptive statistics were used to summarize physician characteristics, reported barriers and facilitators, training needs, and preferred training modalities. Frequencies and percentages were reported for categorical variables, while means and standard deviations were reported for continuous variables. Linear regression was used to identify factors associated with the PrEP implementation training score. Because the training score was derived from the sum of six training topics and approximated a continuous distribution, it was analyzed as a continuous outcome. In the unadjusted model, all five barrier variables and four facilitator variables were entered simultaneously. The adjusted model included all barriers and facilitators, as well as age, gender, race/ethnicity, physician position, clinical specialty, and years in practice. Results are presented as unstandardized regression coefficients (B) and 95% confidence intervals (CI). Statistical significance was defined as *p* < .05.

### Ethical Approval

Ethical approval was obtained from the Louisiana State University Health Science Shreveport Institutional Review Board (IRB) (approval #00002527). The requirement for written informed consent was waived due to the minimal-risk nature of the anonymous survey. No personally identifiable information was collected, and all responses were stored on secure institutional servers accessible only to authorized study personnel.

## Results

### Physician Characteristics

The analytic sample included 100 physicians. The mean age was 36.2 years (SD = 11.8). Most participants were female (57.0%) and White (66.0%). Residents comprised the largest proportion of respondents (63.0%), followed by faculty physicians (27.0%) and fellows (10.0%). Internal medicine was the most frequently represented specialty (26.0%), followed by emergency medicine (18.0%), obstetrics and gynecology (16.0%), family medicine (15.0%), pediatrics (14.0%), and other specialties (11.0%). Most physicians had been in practice for fewer than 5 years (67.0%), while 16.0% reported more than 20 years of clinical experience (see Table 1).

**Table 1:** Characteristics, perceived barriers, facilitators, and training preferences among physicians (*N* = 100)

Variable	*n* (%)
*Respondent characteristics*	
Age, years, mean (SD)	36.2 (11.8)
Gender	
Male	43 (43.0)
Female	57 (57.0)
Race/ethnicity	
White	66 (66.0)
Non-White	34 (34.0)
Position	
Faculty	27 (27.0)
Resident	63 (63.0
Other, includes fellows	10 (10.0)
Specialty	
Internal Medicine	26 (26.0)
Emergency Medicine	18 (18.0)
Pediatrics	14 (14.0)
Family Medicine	15 (15.0)
Obstetrics and Gynecology	16 (16.0)
Other	11 (11.0)
Years in practice	
< 5 years	67 (67.0)
5–10 years	13 (13.0)
11–20 years	4 (4.0)
> 20 years	16 (16.0)
*Outcome*	
PrEP implementation training score, mean (SD)	4.0 (1.5)
*Barriers*	
Lack of training	84 (84.0)
Lack of clinical protocols	70 (70.0)
Lack of laboratory monitoring knowledge	64 (64.0)
Staffing and time constraints	69 (69.0)
Insurance and cost knowledge	72 (72.0)
*Facilitators*	
Protocols and guidelines	92 (92.0)
Peer support	91 (91.0)
Pharmacist support	89 (89.0)
EMR order sets	89 (89.0)
*Preferred training topics*	
PrEP eligibility	89 (89.0)
Side effects and toxicity	89 (89.0)
Contraindications to PrEP	80 (80.0)
Adherence counseling	56 (56.0)
Behavioral risk assessment and sexual history	49 (49.0)
Sexual behavior counseling	42 (42.0)
Other	5 (5.0)
*Preferred training modalities*	
In-person training	60 (60.0)
Self-instructed online courses	48 (48.0)
Online learning resources	41 (41.0)
Clinical consultation with HIV experts	25 (25.0)
Webinar	23 (23.0)
Tele-mentoring with HIV experts	15 (15.0)

^1^SD = standard deviation

^2^PrEP = pre-exposure prophylaxis

### Reported Barriers and Facilitators

Several barriers to PrEP implementation were commonly reported. The most frequently endorsed barrier was lack of provider training or education (84.0%). Other commonly reported barriers included insurance- and cost-related concerns (72.0%), lack of clinical protocols or guidelines (70.0%), staffing and time constraints (69.0%), and lack of knowledge of laboratory monitoring (64.0%) (see Table 1).

Perceived institutional support for PrEP implementation was high. Access to PrEP protocols and guidelines was identified as the most important facilitator (92.0%), followed by peer support from knowledgeable colleagues (91.0%), pharmacist support (89.0%), and EMR order sets with embedded PrEP guidance (89.0%). Additionally, 83.0% of physicians endorsed all four facilitators, suggesting broad support for system-level strategies to enhance PrEP implementation (see Table 1).

### Training Preferences

Most training needs identified by physicians focused on clinical management of PrEP. The most commonly selected training topics were PrEP eligibility criteria (89.0%) and side effects and toxicity (89.0%), followed by contraindications to PrEP use (80.0%). Fewer physicians identified training needs related to adherence counseling (56.0%), behavioral risk assessment and sexual history taking (49.0%), or sexual behavior counseling (42.0%) (see Table 1).

Regarding training delivery, physicians most frequently preferred in-person training (60.0%), followed by self-directed online courses (48.0%) and online learning resources (41.0%). Less commonly preferred formats included clinical consultation with HIV experts (25.0%), webinar-based training (23.0%), and tele-mentoring (15.0%) (see Table 1).

### Predictors of Training Demand

The mean PrEP implementation training score was 4.0 (SD = 1.5). In the unadjusted regression model, endorsement of peer support was associated with a lower training score (B = -1.92, 95% CI: -3.65 to -0.19), whereas endorsement of EMR order sets was associated with a higher training score (B = 1.82, 95% CI: 0.45 to 3.20). No individual barriers were significantly associated with training demand (see Table 2).

**Table 2:** The association between barriers, facilitators, and PrEP implementation training score (*N* = 100)

Predictor	Unadjusted B^1^ (95% CI^2^)	Adjusted B (95% CI)
*Barriers (Ref. = specific item not a barrier)*		
Lack of training	0.76 (-0.08, 1.60)	0.69 (-0.20, 1.58)
Lack of clinical protocols	0.67 (-0.11, 1.24)	0.44 (-0.29, 1.18)
Lack of laboratory monitoring knowledge	0.05 (-0.70, 0.79)	0.06 (-0.72, 0.84)
Staffing and time constraints	-0.14 (-0.89, 0.62)	0.10 (-0.71, 0.92)
Insurance and cost knowledge	0.05 (-0.63, 0.72)	-0.03 (-0.75, 0.68)
*Facilitators (Ref. = specific item not a facilitator)*		
Protocols and guidelines	-0.12 (-1.57, 1.33)	-0.37 (-2.00, 1.27)
Peer support	-1.92 (-3.65, -0.19)^*^	-2.22 (-4.03, -0.41)^*^
Pharmacist support	1.32 (-0.33, 2.97)	1.32 (-0.52, 3.16)
EMR order sets	1.82 (0.45, 3.20)^*^	1.80 (0.27, 3.33)^*^
*Covariates*		
Age, years		-0.06 (-0.14, 0.02)
Gender		
Male		Reference
Female		0.44 (-0.19, 1.07)
Race/ethnicity		
White		Reference
Non-White		-0.26 (-0.98, 0.46)
Position		
Faculty		Reference
Resident		-0.62 (-1.88, 0.65)
Other, includes fellows		-0.82 (-2.27, 0.63)
Specialty		
Internal Medicine		Reference
Emergency Medicine		-0.74 (-1.66, 0.18)
Pediatrics		0.11 (-1.04, 1.26)
Family Medicine		0.46 (-0.53, 1.45)
Obstetrics and Gynecology		-0.61 (-1.63, 0.41)
Other		-0.27 (-1.47, 0.93)
Years in practice		
< 5 years		Reference
5–10 years		-0.31 (-1.42, 0.80)
11–20 years		-0.40 (-2.29, 1.50)
> 20 years		0.74 (-1.65, 3.14)

^1^B = unstandardized regression coefficient.

^2^CI = confidence interval.

^*^p < .05; ^**^p < .01; ^***^p < .001.

In the adjusted model controlling for physician characteristics, both associations remained statistically significant. Physicians endorsing peer support reported lower training scores (B = -2.22, 95% CI: -4.03 to -0.41), whereas those endorsing EMR order sets reported higher training scores (B = 1.80, 95% CI: 0.27 to 3.33). No barriers, demographic characteristics, physician position, specialty, or years in practice were significantly associated with the PrEP implementation training score (see Table 2).

## Discussion

This study was the first in North Louisiana to examine barriers and facilitators to PrEP implementation among physicians at an academic medical center. Despite recognizing PrEP’s importance in HIV prevention, challenges such as insufficient training, insurance concerns, lack of clinical protocols, staffing and time constraints, and limited knowledge of laboratory monitoring hinder routine adoption. Institutional supports like standardized guidelines, peer networks, pharmacist involvement, and EMR order sets could enhance implementation. Physicians expressed interest in further education on eligibility, side effects, toxicity, and contraindications, preferring practical, accessible training formats.

The barriers identified in this study align with findings from prior PrEP implementation research. Lack of provider knowledge and training remains one of the most frequently reported barriers to PrEP prescribing across healthcare settings. In a systematic review of provider-level barriers, Pleuhs et al. [7] found that clinicians commonly reported insufficient familiarity with PrEP guidelines, concerns regarding patient follow-up, and uncertainty regarding prescribing responsibilities. Similarly, Mayer et al. [3] identified inadequate training, concerns regarding adherence monitoring, and insurance-related challenges as persistent barriers to wider PrEP adoption in the United States. More recently, studies conducted among physicians in Texas and across the Southern United States reported that concerns regarding cost, insurance coverage, time constraints, and lack of training continue to hinder PrEP prescribing practices [5, 8].

Although the South accounts for the majority of new HIV diagnoses in the United States, the region continues to experience lower PrEP utilization compared with other regions [1]. Louisiana consistently ranks among the states with the highest HIV diagnosis rates, yet substantial disparities in access to HIV prevention services persist [2]. Structural barriers identified in this study, including limited training opportunities, time constraints, and a lack of standardized clinical protocols, may contribute to the persistent gap between HIV burden and PrEP utilization observed throughout the region. Addressing these implementation barriers may therefore represent an important strategy for strengthening HIV prevention efforts in North Louisiana and similar Southern settings.

An important contribution of this study is the identification of facilitators that providers perceive as supportive of PrEP implementation. The majority of the physicians who participated in the survey said that access to clinical protocols, peer support, pharmacist involvement, and EMR order sets would facilitate PrEP prescription. These findings suggest that physicians do not view PrEP implementation solely as an individual knowledge deficit but rather as a system-level process requiring organizational support. Previous implementation studies have demonstrated that standardized clinical protocols, pharmacist-led interventions, multidisciplinary care teams, and electronic clinical decision-support tools can facilitate PrEP delivery by reducing provider burden and increasing confidence in prescribing practices [10, 11].

Interestingly, endorsement of peer support was associated with lower training demand, whereas endorsement of EMR order sets was associated with higher training demand. Providers with access to knowledgeable colleagues may rely on informal consultation and mentorship, thereby reducing the perceived need for formal training opportunities. In contrast, providers who recognize the value of EMR-based decision-support tools may also be more actively engaged in PrEP implementation efforts and therefore seek additional education to optimize their clinical practice. Although these findings should be interpreted with caution, given the cross-sectional design and modest sample size, they show the potential importance of both interpersonal and technological supports in strengthening implementation readiness.

A significant barrier to implementing PrEP training is that physicians often do not prioritize HIV prevention education due to competing clinical schedules, limited time, and the perception that PrEP falls outside their specialty scope. This pattern has been observed in other studies, with providers reporting insufficient training, uncertainty regarding prescribing responsibilities, and competing clinical demands that limit engagement with PrEP-related education [3, 6–8]. To address these challenges, PrEP education must be embedded in existing educational and clinical frameworks rather than remain optional. Possible approaches include adding PrEP topics to residency programs, CME activities, grand rounds, clinical protocols, and electronic decision-support tools that aid point-of-care decisions [11–13]. Additionally, team-based approaches that leverage pharmacists, nurses, navigators, and other healthcare professionals may help reduce the burden on physicians while expanding access to HIV prevention services [10].

### Limitations

Several limitations should be considered when interpreting these findings. First, this study was conducted at a single academic medical center in Louisiana, which may limit the generalizability of the results to other healthcare settings, including community-based practices, private healthcare systems, and rural clinics. Second, participants were recruited through convenience sampling and voluntary survey participation, creating the potential for selection bias, as providers with greater interest in HIV prevention may have been more likely to participate. Third, all measures were self-reported and may be subject to recall bias or social desirability bias. Fourth, the cross-sectional design precludes drawing conclusions about causality among perceived barriers, facilitators, and training needs. Finally, the sample was predominantly composed of residents and early-career providers, which may not fully reflect the perspectives of more experienced clinicians. Despite these limitations, this study provides important insights into PrEP implementation barriers, facilitators, and workforce training needs within a high HIV-burden region of the Southern United States.

### Practical Implications

The findings of this study suggest several strategies to strengthen PrEP implementation among physicians in high HIV-burden settings. First, PrEP education should be integrated into graduate medical education, continuing medical education (CME), and institutional training programs, with emphasis on practical topics identified by physicians, including eligibility assessment, prescribing, laboratory monitoring, and follow-up care. Flexible training formats that combine in-person sessions with self-paced online modules may improve participation among busy clinicians [7, 14–16].

Second, educational initiatives should be complemented by system-level supports that facilitate PrEP delivery in routine practice. Standardized clinical protocols, EMR-based decision-support tools, and multidisciplinary care models involving pharmacists, nurses, and patient navigators may reduce physician burden and improve implementation [10, 11, 13, 17]. Training should also address common provider-level barriers, including uncertainty regarding prescribing responsibilities, limited time, and discomfort discussing sexual health, while future implementation efforts should evaluate their impact on physician knowledge, confidence, and PrEP prescribing practices [7, 8, 18, 19].

## Conclusion

PrEP implementation remains hindered by gaps in provider training, limited clinical resources, and structural barriers within healthcare systems in the Southern United States. Physicians in this study identified substantial needs for additional education on PrEP eligibility, clinical management, and implementation processes, and emphasized the importance of institutional supports, including clinical protocols, pharmacist involvement, peer support, and EMR-based tools. Findings suggest that successful PrEP implementation requires more than provider education alone and should incorporate system-level strategies that facilitate routine integration into clinical practice. Developing training programs aligned with physicians’ preferences and strengthening organizational support structures may improve PrEP uptake and contribute to ongoing efforts to reduce HIV incidence in Louisiana and other high-burden regions of the South.

## Supplementary Information

Below is the link to the electronic supplementary material.

Supplementary Material 1

## Data Availability

All relevant data are within the manuscript and its Supporting Information files.
